# Prevalence of peripheral artery disease (PAD) and factors associated: An epidemiological analysis from the population-based Screening PRE-diabetes and type 2 DIAbetes (SPREDIA-2) study

**DOI:** 10.1371/journal.pone.0186220

**Published:** 2017-10-26

**Authors:** V. Cornejo del Río, J. Mostaza, C. Lahoz, V. Sánchez-Arroyo, C. Sabín, S. López, P. Patrón, P. Fernández-García, B. Fernández-Puntero, D. Vicent, L. Montesano-Sánchez, F. García-Iglesias, T. González-Alegre, E. Estirado, F. Laguna, C. de Burgos-Lunar, P. Gómez-Campelo, J. C. Abanades-Herranz, J. M. de Miguel-Yanes, M. A. Salinero-Fort, on behalf SPREDIA-2 Group

**Affiliations:** 1 Hospital Carlos III, Madrid, Spain; 2 Grupo de Investigación en cuidados IdIPAZ, Hospital La Paz, Madrid, Spain; 3 Instituto de Investigación Sanitaria del Hospital Universitario La Paz (IdiPAZ), Madrid, Spain; 4 Hospital de Fuenlabrada, Madrid, Spain; 5 Dirección General de Salud Pública, Subdirección de Promoción, Prevención y Educación de la Salud, Consejería de Sanidad, Madrid, Spain; 6 Red de Investigación en servicios de salud en enfermedades crónicas (REDISSEC), Madrid, Spain; 7 Plataforma de Apoyo al Investigador Novel, IdiPAZ, Madrid, Spain; 8 Centro de Salud Monóvar, Madrid, Spain; 9 Hospital Gregorio Marañón, Madrid, Spain; 10 Subdirección General de Investigación Sanitaria, Consejería de Sanidad, Madrid, Spain; Nagoya University, JAPAN

## Abstract

**Aim:**

To describe the prevalence of Peripheral Artery Disease (PAD) in a random population sample and to evaluate its relationship with Mediterranean diet and with other potential cardiovascular risk factors such as serum uric acid and pulse pressure in individuals ranged 45 to 74 years.

**Methods:**

Cross-sectional analysis of 1568 subjects (mean age 6.5 years, 43% males), randomly selected from the population. A fasting blood sample was obtained to determine glucose, lipids, and HbA1C levels. An oral glucose tolerance test was performed in non-diabetic subjects. PAD was evaluated by ankle–brachial index and/or having a prior diagnosis.

**Results:**

PAD prevalence was 3.81% (95% CI, 2.97–4.87) for all participants. In men, PAD prevalence was significantly higher than in women [5.17% (95% CI, 3.74–7.11) vs. 2.78% (95% CI, 1.89–4.07); p = 0.014].

Serum uric acid in the upper quartile was associated with the highest odds ratio (OR) of PAD (for uric acid > 6.1 mg/dl, OR = 4.31; 95% CI, 1.49–12.44). The remaining variables more strongly associated with PAD were: Heart rate >90 bpm (OR = 4.16; 95%CI, 1.62–10.65), pulse pressure in the upper quartile (≥ 54 mmHg) (OR = 3.82; 95%CI, 1.50–9.71), adherence to Mediterranean diet (OR = 2.73; 95% CI, 1.48–5.04), and former smoker status (OR = 2.04; 95%CI, 1.00–4.16).

**Conclusions:**

Our results show the existence of a low prevalence of peripheral artery disease in a population aged 45–74 years. Serum uric acid, pulse pressure and heart rate >90 bpm were strongly associated with peripheral artery disease. The direct association between Mediterranean diet and peripheral artery disease that we have found should be evaluated through a follow-up study under clinical practice conditions.

## Introduction

Peripheral arterial disease (PAD) is an important marker of cardiovascular risk [1], and it is an indicator of widespread atherosclerosis in other vascular territories such as the coronary, carotid, and cerebrovascular arteries [2]. The annual mortality rate derived from epidemiological studies of patients with lower extremity PAD is high [3], with a combined event rate for myocardial infarction, stroke, and vascular death of 4% to 5% per year [4].

The ankle–brachial index (ABI) is the ratio of the ankle to brachial systolic blood pressure, and a value of <0.90 indicates the presence of a flow-limiting arterial disease affecting the limb. The accuracy of the ABI for detecting ≥50% stenosis in the leg arteries is high (75% sensitivity and 86% specificity) [5]. The American Heart Association (AHA) Prevention Conference V highlighted the a low ABI is a consistent independent risk factor for cardiovascular events and mortality and recommended its use to detect subclinical PAD [2, 6] to offer early therapeutic interventions to lower the risk of cardiovascular events and mortality The prevalence of PAD ranges between 1.8% and 25% according to the population studied and the cut-off value of the ABI. In advanced countries it has reported to be 3–10% among those aged 40–70 years, and 10–20% among those over 70 years of age [7]. Data from the Multi-Ethnic Study of Atherosclerosis (MESA) showed that the prevalence of PAD was the same in men and women at 3.7%, but borderline values of ABI were significantly higher in women (10.6% vs. 4.3%) [8]. Likewise, the prevalence is higher in certain population subgroups such as diabetic patients [9] and smokers [10].

The Mediterranean Diet (MeDiet) is characterized by daily consumption of fruits, vegetables, legumes, grains, moderate alcohol intake (1–2 glasses/d of wine), a moderate-to-low consumption of red meat, and a high monounsaturated–to–saturated fat ratio [11].

The PREvención con DIeta MEDiterránea (PREDIMED) study [12] showed for the first time under a randomized controlled trial design that a MeDiet supplemented with either extra-virgin olive oil or nuts is useful in the primary prevention of cardiovascular disease (CVD), PAD, atrial fibrillation, and type 2 diabetes mellitus in individuals at high risk. However, few studies carried out under clinical practice conditions have studied the role of MeDiet on PAD, with unselected patients (with and without CVD) and with usual MeDiet consumption.

To date, five population-based studies [13–16] have been conducted in Spain, showing discordant results in PAD prevalence and associated factors, and none of them reported the influence of MeDiet. Furthermore, these studies were carried out in areas where the compliance to MeDiet is higher than in Madrid [17].

Moreover, serum uric acid is an independent risk factor for cardiovascular events [18], but few studies have explored the possible relationship between serum uric acid levels and PAD [19]. This association is plausible given the previous evidence that serum uric acid may affect vascular endothelial function [20], although the association remains controversial [21].

Lastly, pulse pressure (PP; difference between systolic and diastolic pressures) has been included as a predictor of ABI <0.9 in the Spain REASON risk score, and a recently study using the NHANES data [22] has confirmed this issue. Adding pulse pressure to the periodic evaluation of high-risk patients might be a promising PAD surveillance instrument for the community-based population.

The objectives of the present study are to describe the prevalence of PAD in a random population sample and to evaluate its relationship with MeDiet, and with other potential cardiovascular risk factors such as serum uric acid and pulse pressure in individuals older than 45 years.

## Material and methods

### Design

This study was conducted as part of a broader project, the **S**creening **PRE**-diabetes and type 2 **DIA**betes (SPREDIA-2) study, which has been described in detail elsewhere [23]. SPREDIA-2 is a population-based prospective cohort study in which baseline screening was performed from July 2010 to March 2014.

### Subjects

A total of 2,553 subjects were contacted. Potential participants were selected randomly from the electronic health records of all patients with health care coverage from two districts in the north metropolitan area of Madrid (Spain), namely, Fuencarral-El Pardo and Tetuán, which include three and seven primary health care centers, respectively. Of the 1,592 subjects (62.4%) who agreed to participate, 166 had been diagnosed with DM.

Those subjects not interested in participating were asked to report voluntary sociodemographic and clinical data, which revealed no significant differences in age, sex, or BMI. However, subjects in the participants group had a significantly greater family history of DM, hypertension and dyslipidemia compared with the non-participants group (S1 Table). The study procedure has been described in detail elsewhere [23]. Briefly, recruitment was divided into three phases. First, the potential participants were sent a letter signed by their general practitioner explaining the objectives of the study and inviting them to participate. Second, subjects were contacted by phone to resolve doubts, and, if they were interested in participating, were given an appointment for the assessment. To minimize the losses attributable to failure to locate the patient, up to four telephone calls were made at different times and on different days. Third, the patient attended the assessment in the outpatient clinic of Carlos III Hospital after an overnight fast. Upon arrival, a fasting blood analysis was obtained by measuring blood levels of glucose, creatinine, serum uric acid, HbA1c, serum insulin, and lipids and lipoproteins. Immediately after blood sampling, all subjects with no previous diagnosis of diabetes underwent an oral glucose tolerance test (OGTT) with 75 g of anhydrous glucose in a total fluid volume of 300 ml. A second blood sample was obtained 2 hours later.

### Variables

The ABI values were measured by nurses trained at the Section of Internal Medicine of The Carlos III Hospital and according to current recommendations [24]. The ABI measurements were performed with a bidirectional portable echo-Doppler of 8 MHz (Minidoppler HADECO ES-100, Kawasaki, Japan) and a calibrated sphygmomanometer. The systolic blood pressure (SBP) was measured in the posterior tibial and pedal arteries of both lower limbs and the brachial artery of both upper limbs. The value of the ABI for each limb was calculated dividing the greater SBP obtained in each limb by the SBP of whichever was the higher in the upper limbs. The lowest value obtained was considered the ABI for that individual.

Sociodemographic variables, family history of prevalent diseases, cardiovascular risk factors (smoking habit, hypertension, diabetes, and hypercholesterolemia), clinical history of cardiovascular disease (CVD), comorbidities, and current treatments were recorded for all individuals. Participants were considered as hypertensive when the arterial pressure was ≥140/90 mmHg. Hypercholesterolemia was defined as having LDL-cholesterol ≥100 mg/dl (2.57 mmol/l) and/or receiving hypolipidemic medication. The smoking habit included all who had consumed tobacco over the previous month. A diabetes diagnosis was established when baseline glucose was ≥7 mmol/l (126 mg/dl) on two different occasions, or if the patient was receiving oral hypoglycemic drugs or insulin. CVD included documented history of coronary heart disease (acute myocardial infarction, angina, coronary revascularization procedure), ischemic or hemorrhagic stroke, and PAD. All participants had a physical examination with determination of height, weight, waist circumference (midway between the lowest rib and the iliac crest), and blood pressure. PP was calculated as the difference between systolic and diastolic pressures.

A previously validated 14-item MeDiet Assessment Tool was the method for assessing adherence to the MeDiet [25] where subjects were asked for their consumption of the most common Mediterranean foods (S2 Table). The total score ranges from 0 to 14. Each item was scored 0 (non-compliant) or 1 (compliant) [26]. Higher scores reflected better adherence. High adherence was defined as meeting at least 11 of the 14 items [27].

Cholesterol and triglycerides were determined by enzymatic assays. Low-density lipoprotein cholesterol (LDL-cholesterol) was calculated according to the Friedewald formula (LDL-cholesterol = total cholesterol—([high-density lipoprotein cholesterol (HDL-cholesterol) + triglycerides/5]) in subjects with triglycerides below 400 mg/dl. HDL-cholesterol was measured after precipitation of apo-B lipoproteins. Glucose was measured by the glucose oxidase method. HbA1c was measured by a high-performance liquid chromatography (HPLC) method. Uric acid was measured by the uricase method.

### Statistical analysis

The quantitative variables are presented as means with standard deviation, and the qualitative variables are presented as percentages. To check for normality of distribution of quantitative variables the Kolmogorov-Smirnov test was applied. Comparison of categorical variables was performed using chi-squared tests, and for continuous variables, the ANOVA test was used. The chi-square test for linear trend was used for ordinal variables. To explore associations across the range of ABI levels, ABI was categorized into 4 levels, with <0.9 or known PAD as the lowest level, and tertiles of ABI (0.90–1.09, 1.10–1.19 and ≥1.20, respectively). Logistic regression analyses were performed to evaluate the independent association of PAD with those variables that, in the univariate analysis, showed significance levels of P<0.10, as well as those that were considered clinically important or potentially confounding, such as gender and age. In the fully adjusted analysis, the interaction between gender and age was not significant. The magnitude association was expressed with the Odds Ratio. In all cases, the accepted level of significance was 0.05 or less, with a 95% Confidence Interval (95% CI).

Statistical processing of the data was performed with SPSS v.19 software (IBM Inc, Armonk, NY, USA).

### Ethical considerations

The study protocol had been approved by the Research Ethics Committee of the Carlos III Hospital in Madrid. The study complied with the International Guidelines for Ethical Review of Epidemiological Studies (Geneva, 1991). All patients signed an informed consent form.

## Results

A total of 1,592 subjects agreed to participate in the study, 684 (43%) of whom were male. We excluded 6 participants who did not complete the ABI. Table 1 describes the characteristics of the study sample. From a total of 1,586 patients (mean age 61.5 years), 20 (1.3%) of them were previously diagnosed to have PAD (eight patients showed an ankle-brachial index [ABI] <0.9 and twelve an ABI ≥ 0.9). Out of the 1,566 patients without a previous diagnosis of PAD, 40 (2.5%) showed an ABI < 0.9. The patients previously diagnosed to have PAD with an ABI ≥0.9 (n = 12) did not show differences in cardiovascular risk factors (hypertension, diabetes mellitus, dyslipidemia) or cardiovascular events (coronary artery disease, stroke) compared to individuals with an ABI<0.90, who were defined as having newly diagnosed PAD (n = 48).

**Table 1:** Baseline characteristics of patients included in the study.

	Total N = 1,586
Age (years), mean (SD)	61.5 (6)
Gender (female) (%)	57.0
*Smoking*	
Current smoker (%)	16.1
Former smoker (%)	37.1
Never smoker (%)	46.8
*Level of studies*	
Elementary school (%)	9.7
Junior High school (%)	31.1
Senior High school (%)	27.1
Graduate school (%)	32.2
*History of*	
Hypertension (%)	36.5
Coronary Artery Disease (%)	3.5
Stroke (%)	2.3
Diabetes Mellitus (%)	10.4
Hypercholesterolemia (%)	48.0
*Anthropometries variables*	
Systolic Blood Pressure (mmHg), mean (SD)	124.9 (17)
Diastolic Blood Pressure (mmHg), mean (SD)	77.6 (9.9)
Pulse pressure, mmHg, mean (SD)	47.3 (11.6)
Heart rate, beats/min, mean (SD)	69.2 (10.7)
Waist circumference, cm, mean (SD)	95.1 (12.3)
*BMI*	
BMI <25 Kg/m2 (%)	23.9
BMI 25–29 Kg/m2 (%)	43.9
BMI ≥30 Kg/m^2^ (%)	32.2
Metabolic syndrome (%)	39.5
*Laboratory measures*	
FPG (mg/dl), mean (SD)	105.9 (20.2)
HbA1c (%), mean (SD)	5.80 (0.58)
2h-OGTT (mg/dl), mean (SD)	122.2 (43.0)
2h-OGTT ≥200 mg/dl (%)	5.7
LDL-Cholesterol (mg/dl), mean (SD)	132.8 (33.6)
HDL-Cholesterol (mg/dl), mean (SD)	54.0 (14.6)
Total Cholesterol (mg/dl), mean (SD)	207.4 (37.9)
Triglycerides (mg/dl), mean (SD)	104.2 (69.9)
Serum Uric Acid, mg/dl, mean (SD)	5.27 (1.30)
Creatinine, mg/dl, mean (SD)	0.76 (0.21)
eGFR <60 mL/min/1.73 m2 (%)	1.3
Mediterranean diet score, mean (SD)	8.6 (2.1)
Baseline score for Adherence to Mediterranean Diet >11 (%)	18.7
*Use of*	
Diuretics (%)	13.5
Antiaggregants (%)	9.1
Anticoagulants (%)	1.9
Beta-blockers (%)	7.9
Calcium channel antagonists (%)	5.6
Renin-angiotensin system blockers (%)	25.2
Statins (%)	28.0

BMI: Body Mass Index; FPG: Fasting Plasma Glucose; eGFR: estimated

Glomerular Filtration Rate; OGTT: Oral Glucose Tolerance Test.

The patients previously diagnosed of PAD had a high morbidity in comparison with non-PAD patients [25% coronary artery disease vs. 3.4% (p<0.001), higher prevalence of diabetes mellitus (41.7% vs. 10.2%; p<0.001), hypercholesterolemia (75% vs. 47.8%; p = 0.06)], were more frequently treated with antiaggregants (41.7% vs. 8.8%; p<0.001) and their adherence to MeDiet was moderately higher (25% vs. 18.7%; p = 0.58).

**Fig 1:**
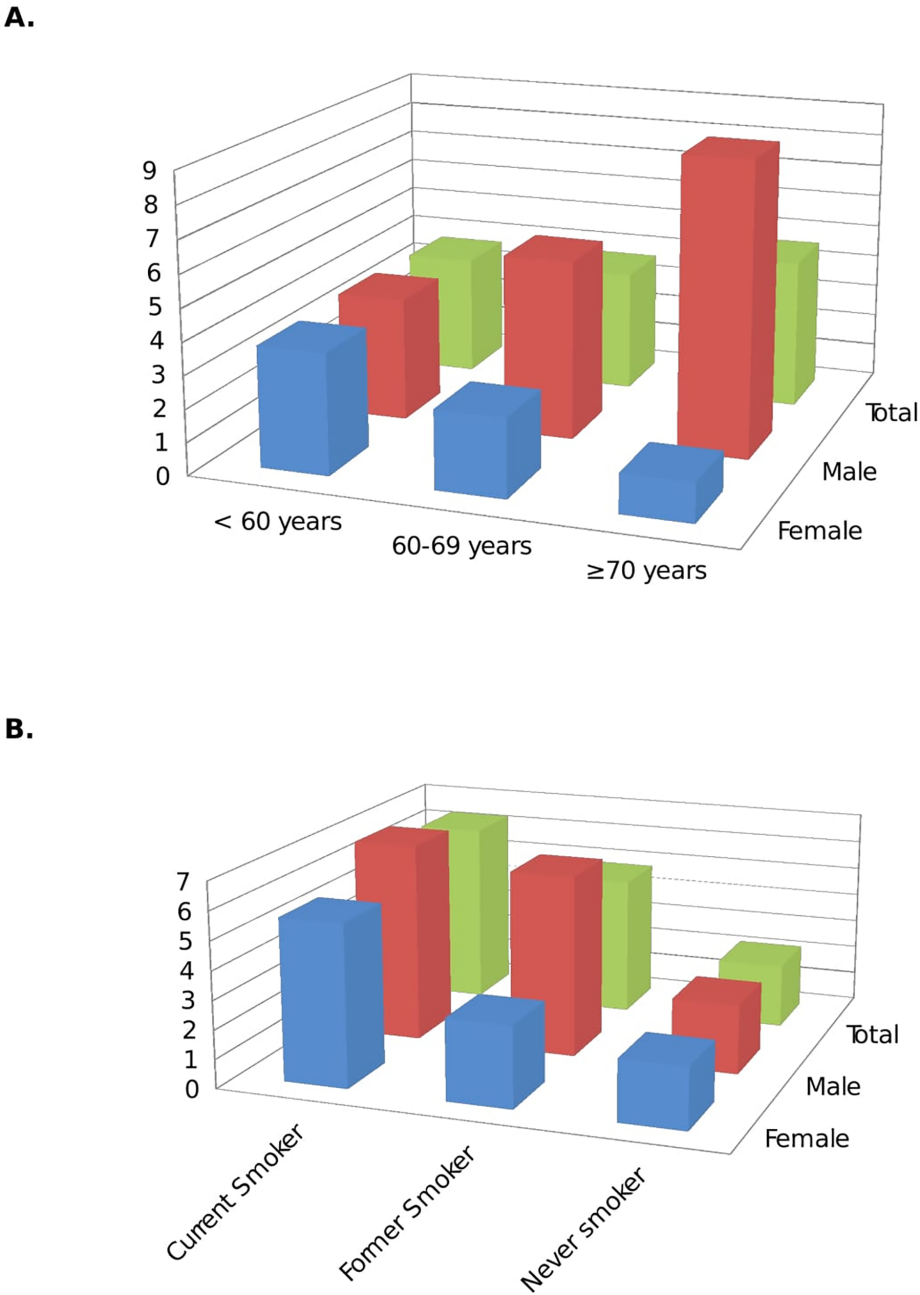
Prevalence of PAD stratified by age group and gender (A) and by smoking status and gender (B).

**Fig 2:**
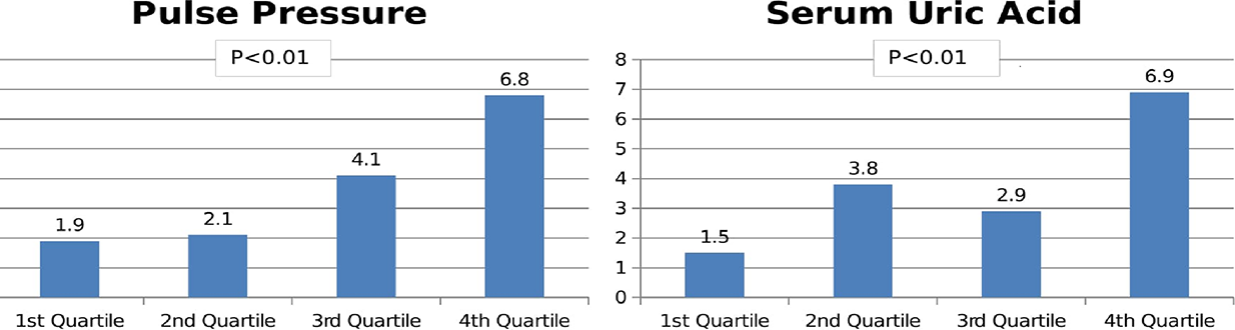
Prevalence of peripheral artery disease by quartile of pulse pressure and serum acid uric.

PAD prevalence was 3.81% (95% CI, 2.97–4.87) for all participants. In men the PAD prevalence was significantly higher than in women [5.17% (95% CI, 3.74–7.11) vs. 2.78% (95% CI, 1.89–4.07); p = 0.014]. The prevalence of PAD increased with age in men, from 3.8% in subjects aged <60 years to 9% in those aged ≥70 years. In women, however, the prevalence decreased from 3.7% in subjects aged <60 years to 1.2% in those aged ≥70 years (Fig 1A). Furthermore, the prevalence of PAD was lower in never-smokers than in current or former smokers (Fig 1B). Also, for former smokers the PAD prevalence in men was 2.25-fold greater than in women (6.3% vs. 2.8%), but we did not find large differences in the PAD prevalence for the current smoker population between both genders. We found positive associations between PAD and PP and serum uric acid values grouped in quartiles (Fig 2).

Patients in lower ABI categories were more likely to be older and male and to have a higher number of traditional cardiovascular disease risk factors (Table 2).There were statistically significant differences between ABI categories in relation to: smoking status, level of studies, coronary artery disease, hypertension, diabetes, hypercholesterolemia, systolic blood pressure, PP, heart rate, waist circumference, metabolic síndrome, HbA1c, serum uric acid, creatinine, and use of diuretics, beta-blockers, antiaggregant, renin-angiotensin system blockers and statins.

In the univariate analysis, the OR of PAD for male gender was 1.91 (95% CI, 1.13–3.22; p = 0.014). However, after fully adjusting for all covariates used in our analyses, the OR changed to a non-significant 1.30 (95% CI, 0.69–2.57; p = 0.40) for the female gender.

**Table 2:** Demographic and clinical characteristics by ankle-brachial index (ABI) category.

	PAD/ABI<0.90 n = 60	ABI 0.90–1.09 n = 498	ABI 1.10–1.19 n = 503	ABI ≥ 1.19 n = 525	p-value*
Age (years), mean (SD)	62.4 (6)	61.9 (6)	61.5 (5.9)	61.4 (6)	0.023
Gender (female) (%)	41.7	73.7	57.5	42.5	<0.001
*Smoking*					
Current smoker (%)	26.7	17.1	14.6	15.4	0.018
Former smoker (%)	46.7	33.3	37.9	38.7	
Never smoker (%)	26.7	49.6	47.5	45.9	
*Level of studies*					
Elementary school (%)	8.6	13.7	10.6	5.1	<0.001
Junior High school (%)	41.4	31.3	29.7	31.0	
Senior High school (%)	27.6	29.7	28.7	23.0	
Graduate school (%)	22.4	25.3	31.1	40.8	
*History of*					
Hypertension (%)	51.7	39.2	33.8	34.7	0.017**
Coronary Artery Disease (%)	15	4.6	2.8	1.9	<0.001**
Stroke (%)	1.7	2.2	2.6	2.3	0.962
Diabetes Mellitus (%)	21.7	12.0	10.3	7.6	0.001**
Hypercholesterolemia (%)	58.3	50.5	47.2	45.1	0.024**
*Anthropometries variables*					
Systolic Blood Pressure (mmHg), mean (SD)	132.6 (18)	125.8 (17)	124.4 (17)	123.6 (17)	0.001
Diastolic Blood Pressure (mmHg), mean (SD)	78.9 (9.9)	78.1 (9.7)	77.7 (10.5)	76.8 (9.7)	0.123
Pulse pressure, mmHg, mean (SD)	53.7 (12.7)	47.6 (12.5)	46.7 (10.8)	46.8 (11)	<0.001
Heart rate, beats/min, mean (SD)	71.9 (14)	69.7 (10.7)	69.4 (10.6)	68.2 (10.2)	0.025
Waist circumference, cm, mean (SD)	100.1 (12.8)	93.9 (12.7)	94.6 (11.8)	96.1 (12.1)	<0.001
*BMI*					
BMI <25 Kg/m2 (%)	16.7	24.5	27.0	21.1	0.063
BMI 25–29 Kg/m2 (%)	41.7	41.2	42.1	48.4	
BMI ≥30 Kg/m^2^ (%)	41.7	34.3	30.8	30.5	
Metabolic syndrome (%)	60.0	41.8	38.8	35.8	0.001**
*Laboratory measures*					
FPG (mg/dl), mean (SD)	112.8 (26)	105.7 (20.5)	105.8 (20.4)	105.5 (18.8)	0.066
HbA1c (%), mean (SD)	6.07 (0.69)	5.86 (0.64)	5.80 (0.56)	5.72 (0.51)	<0.001
2h-OGTT (mg/dl), mean (SD)	132.2 (53.5)	120.9 (42.1)	120.5 (39.1)	123.9 (45.9)	0.214
2h-OGTT ≥200 mg/dl (%)	10.6	5.6	4.5	6.5	0.272
LDL-Cholesterol (mg/dl), mean (SD)	134.5 (38.3)	133.0 (34.8)	133.7 (33.0)	131.5 (32.5)	0.722
HDL-Cholesterol (mg/dl), mean (SD)	51.5 (12.3)	54.9 (14.8)	54.7 (14.8)	52.9 (14.4)	0.060
Total Cholesterol (mg/dl), mean (SD)	209 (41)	208.8 (39.6)	208.2 (36.9)	205.1 (36.9)	0.391
Triglycerides (mg/dl), mean (SD)	115.7 (67.4)	106.9 (78.1)	100.7 (58.8)	103.8 (71.5)	0.318
Serum Uric Acid, mg/dl, mean (SD)	5.76 (1.4)	5.09 (1.3)	5.21 (1.28)	5.45 (1.31)	<0.001
Creatinine, mg/dl, mean (SD)	0.83 (0.39)	0.72 (0.18)	0.75 (0.19)	0.79 (0.21)	<0.001
eGFR <60 mL/min/1.73 m2 (%)	5.0	1.0	1.4	1.1	0.080
Mediterranean diet score, mean (SD)	8.8 (2.5)	8.6 (2.1)	8.7 (2.1)	8.6 (2.1)	0.657
Baseline score for Adherence to Mediterranean Diet >11 (%)	31.7	17.5	19.1	18.0	0.062
*Use of*					
Diuretics (%)	25.0	15.1	12.1	12.0	0.015**
Antiaggregants (%)	28.3	10.1	7.8	7.2	<0.001**
Anticoagulants (%)	1.7	2.2	1.8	1.7	0.938
Beta-blockers (%)	16.7	8.2	8.3	6.1	0.023**
Calcium channel antagonists (%)	10.0	5.6	4.4	6.1	0.273
Renin-angiotensin system blockers (%)	40.0	26.0	23.5	24.4	0.044
Statins (%)	45.0	31.3	27.8	23.0	<0.001**

*p value for comparison between 4 categories of ABI (ANOVA test or Chi-squared test for means or proportions respectively).

**p value for linear trend (Chi-squared test for linear trend).

BMI: Body Mass Index; FPG: Fasting Plasma Glucose; eGFR: estimated Glomerular Filtration Rate; OGTT: Oral Glucose Tolerance Test

Table 3 shows the results of the individual analysis of the risk factors associated with the presence of PAD according to the multivariate logistic regression model adjusted for age, gender, and those variables with a p-value of less than 0.10 in the univariate analysis. Serum uric acid in the upper quartile was associated with the highest OR of PAD (for uric acid > 6.1 mg/dl, PR = 4.31; 95% CI, 1.49–12.44). The remaining variables more strongly associated with PAD were: Heart rate >90 bpm (OR = 4.16; 95%CI, 1.62–10.65), PP in the upper quartile (≥ 54 mmHg) (OR = 3.82; 95%CI, 1.50–9.71), adherence to MeDiet (OR = 2.73; 95% CI, 1.48–5.04), and former smoker status (OR = 2.04; 95%CI, 1.00–4.16).

**Table 3:** Factors associated with peripheral artery disease (logistic regression analysis).

	PR	95% CI OR	p-value
**Gender**			
Male	1		
Female	1.301	0.661–2.562	0.446
**Age** (per unit of increment)	0.989	0.939–1.043	0.687
**HbA1c** (per unit of increment)	1.371	0.863–2.178	0.181
**Antiaggregants**			
No	1		
Yes	2.373	0.983–5.727	0.055
**eGFR <60 mL/min/1.73 m**^**2**^			
No	1		
Yes	2.178	0.526–9.027	0.283
**Adherence of Mediterranean diet**			
No	1		
Yes	2.729	1.479–5.036	0.001
**Diuretics**			
No	1		
Yes	1.985	0.907–4.342	0.086
**Coronary Artery Disease**			
No	1		
Yes	1.971	0.592–6.567	0.269
**Smoking**			
Never smoker	1		
Current smoker	4.069	1.841–8.995	0.001
Former smoker	2.041	1.001–4.162	0.049
**Serum Uric Acid**			
1^st^ Quartile	1		
2^nd^ Quartile	2.503	0.912–6.870	0.075
3^nd^ Quartile	1.667	0.546–5.088	0.369
4^th^ Quartile	4.314	1.496–12.446	0.007
**Pulse pressure**			
1^st^ Quartile	1		
2^nd^ Quartile	1.201	0.411–3.508	0.738
3^nd^ Quartile	2.088	0.839–5.196	0.113
4^th^ Quartile	3.819	1.502–9.709	0.005
**Heart rate >90 beats/min**			
No	1		
Yes	4.161	1.625–10.650	0.003

Adjusted for Diabetes (known and unknown), Arterial Hypertension, Metabolic syndrome

Beta blockers, Renin-angiotensin system inhibitors and Statins use.

OR: Odds Ratio; CI: Confidence Interval; eGFR: estimated Glomerular Filtration Ratio.

## Discussion

The results of our study show that the prevalence of PAD is low in comparison with other international population-based studies [28], but similar to that found in the Hermex Study carried out in Badajoz (Spain) [29]. A recent systematic review for Peripheral Arterial Disease Research Coalition including 34 community-based studies [30] evidenced that prevalence of PAD ranged between 7.3–11.8% for women aged 50–74 years and between 6.4–12.1% for men aged 50–74 years among high-income countries. Spain belongs to the high-income countries category, and for this reason, one might expect a higher prevalence of PAD. However, a “Spanish paradox” has been described as a phenomenon by which the cardiovascular morbidity (myocardial infarction stroke and PAD) and mortality levels are dissociated from their cardiovascular risk factors. The existence of protector factors such as MeDiet and its interaction with different genetic patterns [31] has been argued as a plausible explanation of this phenomenon.

Furthermore, other Spanish population-based studies have shown PAD prevalence ranging from 4.5% [13] to 10.5% [16]. The prevalence was higher in men than in women for all Spanish studies. Nevertheless, some community-based studies have shown a higher prevalence of PAD in women compared with men [32–35], even in each decade of life [33]. These findings raise concerns about whether there should be differences in the definition of normal ABI values between men and women, and therefore whether the diagnostic criteria of PAD should be based on a cut-off ABI value different to the currently accepted as standard.

It is commonly accepted that men have a higher prevalence of PAD than women until the seventh decade of life [16, 29, 36]. However, in our study, the PAD prevalence was lower in women in comparison with men for each age group. Also, we found an inverse relationship between PAD and advancing age among women. We have no strong explanation for this finding, in which chance could indeed be playing a role.

The direct association between adherence to MeDiet and PAD is an unexpected phenomenon, given the strong evidence of the reduction of risk of PAD with the daily consumption of MeDiet [37]. Some aspects may explain our results. Firstly, the PREDIMED Randomized Trial compared two groups of MeDiet supplemented with extra-virgin olive oil and nuts, respectively, with a group who received counseling on a low-fat diet (control group), and all groups received a comprehensive dietary educational program based on individual and group sessions with a dietitian every 3 months designed to increase adherence to the MeDiet or a low-fat diet. The use of the 14-point MeDiet questionnaire proved very useful because the results formed the basis for personalized advice on changes the participant should make to acquire a traditional MeDiet or low-fat diet pattern. However, in the present study, no person received counseling and/or supplements of diet. Quite simply, we merely asked them for their consumption of MeDiet using the 14-point MeDiet questionnaire. These differences might help explain our findings. Secondly, as it is well known, a cross-sectional study like ours does not allow the establishment of a causal relationship between MeDiet and PAD. Thus, patients with baseline known PAD might have initiated MeDiet as part of their treatment, given that these individuals with known PAD had high vascular morbidity and a trend to show a better adherence to MeDiet compared with those without PAD. Thirdly, is plausible that those patients with known PAD and those with compatible symptoms of PAD (i.e. intermittent claudication) would tend to conserve healthier diets like the MeDiet whereas the population with a good perception of their health would lead to a progressive abandonment of the MeDiet, due to the economic crisis. This phenomenon has been detected in Italy, with a dramatic fall in the adherence to MeDiet, lowering the adherence from over 30% to 18% in the whole population with the global economic crisis [38]. This hypothesis might partially explain the results obtained here.

The positive association between smoking status and PAD is well established, and it is habitually found in the vast majority of studies [14, 16, 30, 34]. Our study is consistent with these findings, but shows slight differences in the PAD prevalence between current and former smokers in the case of men. These findings are concordant with a recent systematic review of 34 studies [30]. However, other studies in our country showed greater differences between both levels of smoking status [14].

Our findings of a strong, independent association between serum acid uric >6.1 mg/dl and PAD is congruent with previous studies in adults with high cardiovascular risk [39, 40] and among the general population [41]. Serum uric acid has been found to be associated with several inflammatory markers, including C-reactive protein and interleukin-6 [42]. Furthermore, hyperuricemia has been reported as a factor responsible for cardiovascular diseases through endothelial dysfunction caused by inactivation of nitric oxide, which is a potent vasodilator [43], and arresting the proliferation of endothelial cells [44].

However, it is well known that there is a high correlation between serum uric acid and glomerular filtration rate (GFR), strengthening the possibility that Chronic Kidney Disease (CKD) status may be a statistical confounder in the relationship between uric acid and cardiovascular disease, rather than the mediator [45]. In recent years, a better understanding of uric acid metabolism suggests that CKD may be an intermediate step between hyperuricemia and cardiovascular disease, and increased levels of uric acid are, at once, a dependent and independent risk factor of cardiovascular disease and kidney disease progression [46].

As large-artery stiffness increases in middle-aged and elderly subjects, SBP rises, and DBP falls, with a resulting increase in PP [47]. A series of prospective and cross-sectional studies have shown that PP is associated with cardiovascular events [47–49] and mortality [47, 50–51]. Interest has been increasing on the association between PP and PAD. In this line, previous studies have shown an association between PP and PAD [52–54]. Our results are in accordance with these findings, but our study was carried out in a general population rather than a population with a high risk of cardiovascular disease as other studies [55,56]. The Multi-Ethnic Study of Atherosclerosis based in subjects free of cardiovascular disease has shown a tendency, though not statistically significant, to a higher proportion of patients with PAD for each 10 mmHg increase in PP [57].

To our knowledge, this is the second study that has shown an association between high heart rate and PAD. In The MERITO Study [58], for each increase in the heart rate of one beat per minute, the OR for PAD was 1.02 (96% CI, 1.01–1.03). Resting heart rate has been associated with all-cause and cardiovascular mortality [59,60]. Some studies have found indirect associations between heart rate and PAD. So, resting heart rate ≥77 beats/min has been associated with frailty in older men (age-adjusted OR = 1.90; 95%CI, 1.30–2.48) [61] and frailty is strongly associated with subclinical PAD (ABI<0.8) (OR = 3.56; 95%CI, 2.03–6.24) [62].

A practical application of our findings is that heart rate>90 beats/min and a serum uric acid above 6.1 mg/dl could be two factors to consider in selecting patients to screen for PAD, regardless of gender, age and diabetes status. Nevertheless, this proceeding requires caution because the pathophysiological basis of the relationship between resting heart rate and PAD is still unknown.

Other known factors firmly established to increase the risk of PAD, such as diabetes mellitus, hypertension, or hypercholesterolemia were not associated in the multivariate analysis with the presence of a low ABI. This is a phenomenon already observed in other studies [58, 63, 64], and may be due to the known limitations of cross-sectional studies, the lack of a sufficient number of cases of the disease or the exclusion of persons with an ABI >1.5, who are more likely to have diabetes mellitus.

Concerning to the limitations of the study, the cross-sectional design did not allow determination of the causal effect of variables studied with PAD. Also, women were more likely to participate than men, as usually occurs in population-based studies. This aspect would limit the inference of the results to the entire population. Also, our findings about the magnitude of the association between both serum uric acid and high heart rates with PAD could have been affected by a potential residual confounding by unknown or misspecified confounding variables.

The study presents some strengths consisting of having collected epidemiological information on the prevalence of PAD in a sample of a wide range of ages, representative of the general population and from a region of our country with high prevalence of risk factors. Having used the same methodology as in other published population-based studies allows comparability to these.

In conclusion, our results demonstrate the existence of a low prevalence of PAD in a population aged 45–74 years. Serum uric acid, pulse pressure and heart rate >90 bpm were strongly associated with PAD. The direct association between MeDiet and PAD that we have found should be further evaluated through a follow-up study under clinical practice conditions.

## Supporting information

S1 Table Differences between participants and non-participants at recruitment phase. (DOCX)

S2 Table Validated 14-item Questionnaire of Mediterranean diet adherence. (DOCX)
