# Supplementary material for: Prevalence of peripheral artery disease (PAD) and factors associated: An epidemiological analysis from the population-based Screening PRE-diabetes and type 2 DIAbetes (SPREDIA-2) study
Source: PLoS One. 2017 Oct 26;12(10):e0186220. doi: 10.1371/journal.pone.0186220 (PMC5657631; doi:10.1371/journal.pone.0186220)
Supplement: S1 Table — (DOCX) [file pone.0186220.s001.docx]

|  |  |  |  |  |
| --- | --- | --- | --- | --- |
|  | **Participants** | **Non participants** | | **p-value** |
| **Female, gender** % | 58.3 | 56.5 | | 0.367 |
| **Age**, *mean (SD)* | 61.7 (6) | 61.3 (6.2) | | 0.103 |
| **Family history of DM**, % | 30.6 | 25.7 | | < 0.01 |
| **Hypertension,** % | 34.9 | 22.5 | | <0.01 |
| **Dyslipidemia,** % | 42.9 | 28.0 | | <0.01 |
| **BMI Kg/m^2^,** *mean (SD)* | 28.2 (4.7) | 27.9 (4.1)* | | 0.090 |

*Calculated from Weight and Height self-reported
