# Supplementary material for: Prevalence of peripheral artery disease (PAD) and factors associated: An epidemiological analysis from the population-based Screening PRE-diabetes and type 2 DIAbetes (SPREDIA-2) study
Source: PLoS One. 2017 Oct 26;12(10):e0186220. doi: 10.1371/journal.pone.0186220 (PMC5657631; doi:10.1371/journal.pone.0186220)
Supplement: S2 Table — (DOCX) [file pone.0186220.s002.docx]

| **Questions** | **Criteria for 1 point** |
| --- | --- |
| 1. Do you use olive oil as main culinary fat? | Yes |
| 2. How much olive oil do you consume in a given day (including oil used for frying, salads, out-of-house meals, etc.)? | ≥ 4 tbsp |
| 3. How many vegetable servings do you consume per day? (1 serving : 200 g [consider side dishes as half a serving]) | ≥ 2 (≥1 portion raw or as a salad) |
| 4. How many fruit units (including natural fruit juices) do you consume per day? | ≥3 |
| 5. How many servings of red meat, hamburger, or meat products (ham, sausage, etc.) do you consume per day? (1 serving: 100–150 g) | < 1 |
| 6. How many servings of butter, margarine, or cream do you consume per day? (1 serving: 12 g) | < 1 |
| 7. How many sweet or carbonated beverages do you drink per day? | < 1 |
| 8. How much wine do you drink per week? | ≥ 7 glasses |
| 9. How many servings of legumes do you consume per week? (1 serving : 150 g) | ≥ 3 |
| 10. How many servings of fish or shellfish do you consume per week? (1 serving 100–150 g of fish or 4–5 units or 200 g of shellfish) | ≥ 3 |
| 11. How many times per week do you consume commercial sweets or pastries (not homemade), such as cakes, cookies, biscuits, or custard? | < 3 |
| 12. How many servings of nuts (including peanuts) do you consume per week? (1 serving 30 g) | ≥ 3 |
| 13. Do you preferentially consume chicken, turkey, or rabbit meat instead of veal, pork, hamburger, or sausage? | Yes |
| 14. How many times per week do you consume vegetables, pasta, rice, or other dishes seasoned with sofrito (sauce made with tomato and onion, leek, or garlic and simmered with olive oil)? | ≥ 2 |
